# Mindfulness-based positive psychology interventions: a systematic review

**DOI:** 10.1186/s40359-021-00618-2

**Published:** 2021-08-06

**Authors:** Joshua George Allen, John Romate, Eslavath Rajkumar

**Affiliations:** Department of Psychology, School of Social and Behavioral Sciences, Central University of Karnataka, Kalaburagi, India

**Keywords:** Mindfulness, Eudaimonia, Hedonia, Well-being, MBI

## Abstract

**Background:**

There are hundreds of mindfulness-based interventions in the form of structured and unstructured therapies, trainings, and meditation programs, mostly utilized in a clinical rather than a well-being perspective. The number of empirical studies on positive potentials of mindfulness is comparatively less, and their known status in academia is ambiguous. Hence, the current paper aimed to review the studies where mindfulness-based interventions had integrated positive psychology variables, in order to produce positive functioning.

**Methods:**

Data were obtained from the databases of PubMed, Scopus, and PsycNet and manual search in Google Scholar. From the 3831 articles, irrelevant or inaccessible studies were eliminated, reducing the number of final articles chosen for review to 21. Interventions that contribute to enhancement of eudaimonia, hedonia, and other positive variables are discussed.

**Results:**

Findings include the potential positive qualities of MBIs in producing specific positive outcomes within limited circumstances, and ascendancy of hedonia and other positive variables over eudaimonic enhancement.

**Conclusion:**

In conclusion, exigency of modifications in the existing MBIs to bring about exclusively positive outcomes was identified, and observed the necessity of novel interventions for eudaimonic enhancement and elevation of hedonia in a comprehensive manner.

## Background of the study

Mindfulness, a practice of conscious non-judgmental awareness to the present, emerged in the Indian subcontinent approximately 2500 years ago [1]. Around four decades back, with the pioneering works of Kabat Zinn who had incorporated mindfulness into psychotherapy, the scientific application of mindfulness for health and well-being for specific contextual needs had started [2]. Since then and particularly in the last decade, academic interest in the area of mindfulness has been increasing and the applicability of mindfulness in various facets of life is also getting attention [3–8]. Most of the accessible Mindfulness-Based Interventions (MBIs) are either standalone therapies or facilitating therapies for various clinical disorders and problems. Although mindfulness contributes immensely to clinical psychology, the concept of mindfulness has a broader and vaster meaning, beyond clinical symptom reduction and toward positive human functioning and flourishing. In recent years, Positive Psychology Interventions (PPIs) that integrate mindfulness elements have shown some promising outcomes [1, 9, 10]. Still, there is a significant lack of clarity in the implementation of Mindfulness-Based Positive Psychology Interventions (MPIs) and their impact on positive human functioning. Hence the current study aims to find and analyze the mindfulness-based interventions from the existing literature which have also shown potentials to be a positive psychology intervention.

Beginning from Mindfulness-Based Stress Reduction (MBSR) proposed by Kabat Zinn in the 1970s, MBIs are mostly used in the clinical settings for managing disorders and supporting prognosis of disorders and diseases. As an attempt to balance this reductionist deficit-model of health, the well-being outcomes of MBIs are also studied, especially in recent years. A positive psychology intervention is defined as “an intervention, therapy, or activity, primarily aimed at increasing positive feelings, positive behaviors, or positive cognitions, as opposed to ameliorating pathology or fixing negative thoughts or maladaptive behavior patterns” [12]. In this context, an MPI is “a mindfulness-based intervention with the primary aim to enhance positive human functioning”. PPIs not only exert impact on positive variables, but also are effective among clinical populations—as standalone therapies or facilitating interventions—such as the individuals suffering from depression [11, 12], affective disorders [13], generalized anxiety disorder [14], and eating disorders [15]. Apart from that, regardless of the nature of population, PPIs have the potential to enhance positive cognition, positive affect, positive behavior, and overall positive functioning and experiences. Integrating positive psychology with mindfulness, or accommodating mindfulness elements in psychotherapy, a number of MBIs are developed, however, the existing literature is insufficient to articulate the quality and quantity of the researches on MPIs. In order to fill this gap, research on the current status of MPIs is warranted.

Furthermore, well-being mainly consists of hedonia and eudaimonia, two highly correlated but distinct forms of well-being, with different characteristics [16, 17]. Though they are not mutually exclusive or antagonistic to each other, their nature, intensity, and patterns of expression are singular. This paper attempts to consolidate major mindfulness-based interventions devised for the enhancement of positive functioning beyond clinical symptom reduction, with a given priority to determine the status of MPIs for eudaimonic enhancement. Secondary importance is given to MPIs or MBIs for hedonic well-being and other positive psychology variables such as hope, happiness, resilience, gratitude, flow, compassion, and improved psychological performance. Studies, where a positive psychology variable was just one among three or more dependent variables, were excluded due to their orientation toward the deficit model rather than to positive psychology.

### The positive potentials of mindfulness

#### Eudaimonic enhancement

Eudaimonia is originally a Greek term that can be translated from a subjective perspective as “happiness”, and from an objective point of view, as “flourishing” [18]. Broadly, eudaimonia is “the pursuit, manifestation, and/or experience of virtue, personal growth, self-actualization, flourishing, excellence, and meaning” [19]. The “mindfulness-to-meaning theory” proposed by Garland et al. [20], advocates that mindfulness broadens the awareness spectrum resulting in cognitive-reappraisal of events to include the positive possibilities of specific instances, that enable the individual to perceive the meaning and purpose of life experiences. The cumulative effects of meaningful positive experiences bring about eudaimonic well-being. And unlike hedonia, eudaimonia will gradually expand on its own without the support of any external agencies, and its possibilities are literally infinite. This nature of eudaimonia is elucidated by the concepts of “eudaimonic staircase” [21] and the “upside spiral of positive emotions” described in the *broaden-and-build theory* [22]. Thus, this review is expected to be a beneficial contribution to the existing scientific knowledge on the role of MBIs in utilizing the eudaimonic-enhancement capability of mindfulness. In addition, recognition of the current utility spectrum of MBIs would support better usage of those MBIs as eudaimonic enhancement tools, or signify the need of further explorations on MBIs for eudaimonic enhancement.

#### Hedonic enhancement

Hedonia or hedonic well-being shall be defined as “the pursuit and/or experience of pleasure, enjoyment, comfort, and reduced pain” [19]. Since pain reduction is an element of hedonia, all kinds of psychotherapies are, in a sense, involved in the augmentation of hedonic well-being. Along with eudaimonia, hedonia contributes to subjective well-being [18]. The efficacy of MBIs is associated with fulfillment of hedonic needs. Some studies have explored the relationship between mindfulness and hedonic well-being and confirmed the assumption that mindfulness functions in direct and indirect ways to induce pleasure and reduce pain [23, 24]. Other than clinical symptom management, hedonia and its components can be induced by MBIs, independently as well as in combination with other positive variables. Enhancement of enjoyment [25], happiness [26], and positive affect [27, 28] are a few examples. Although not always directly stated, improved hedonia—reduced pain and discomfort or improved pleasure and comfort—had been described in clinical literature where MBIs are utilized for therapeutic purpose. The existing literature recognizes the hedonic enhancement quality of mindfulness, but they are not being studied comprehensively. Hence, this review gives auxiliary importance to narrate the MBIs that produced hedonic well-being.

#### Increasing other positive outcomes

Theoretically and empirically, mindfulness is found to be connected with a number of positive psychology variables. Different MBIs often focus on a specific aspect such as compassion, relaxation, and cognitive skills. Literature suggests that MBIs are effectual in generating a number of positive outcomes such as hope [29], optimism [30, 31], prosocial behavior [32], flow [33] working memory [34], and academic performance [35]. This paper also reviews MBIs that had produced positive outcomes in addition to eudaimonic and hedonic well-being, in expectation of identifying the extent of impact conceivable for an MPI.

## Purpose of the study

This paper attempts to present a narrative/descriptive synthesis of the major MBIs with positive potentials. Firstly, it intends to identify standardized or empirically validated MPIs. Secondly, MBIs that produce positive functioning shall be recognized and their efficacy as an MPI will be verified. Further, the MBIs that improve hedonic well-being and/or other positive variables will also be reported. Finally, the study stands to recount the intention of MBIs in eudaimonic enhancement.

## Methods

Data were drawn from three electronic databases—PubMed, Scopus, and PsycNet—and a manual search in Google Scholar, from the inception to 29 May 2020. Keyword string used for database search was “mindfulness intervention” and filters were “controlled clinical trials” and “randomized controlled trial” in PubMed; “articles” and “psychology” in Scopus; and “articles” in PsycNet.

1. Eligibility criteria

The inclusion criteria were: (i) studies with the application of mindfulness-based intervention regardless of the population characteristics such as age, gender, and ethnicity; (ii) experimental and quasi-experimental studies that compared the outcomes between individuals administered with and without an MBI; and (iii) studies with positive psychology variables as dependent variables. The exclusion criteria followed to eliminate the articles were: (i) review papers, (ii) medical/ neuropsychological researches, and (iii) studies with positive psychology variables as just one among three or more dependent variables.

Positive psychology outcomes considered included but not limited to general well-being, eudaimonic well-being, hedonic well-being, happiness, hope, grit, loving kindness, gratitude, empathy, and flourishing. Studies where positive psychology variable was just one among the three or more dependent variables were excluded. It was because the focus of the current research was to find the MBIs that produced positive psychology variables as outcomes (or positive outcomes); and due to the dichotomous nature of many psychological variables, they have a positive and negative continuum which can be reported as the presence or absence of either positive or negative end. If three or more dependent variables are assessing clinical or non-positive conditions, it is highly likely that the one positive variable among these is the absence of a clinical condition rather than a positive psychology outcome. For instance, well-being is often reported as the absence of a clinical condition such as anxiety or depression. Also, when majority of the outcome measures are related to non-positive variables, the intervention is less likely to be developed for positive impacts. Including such studies would redirect the focus of the study and unnecessarily increase the time, energy, and resources for conducting the research.

Review papers were excluded because the study focused on original researches that reported outcomes of an MBI. Papers on medical/ neuropsychological researches were also excluded because their focus was not identification of positive psychology variables as the outcome measures of MBIs. Rather than the physiological mechanisms behind exposure to an MBI, the current study focused on perceived enhancement of positive psychology variables.

2. Data collection

**Fig. 1:**
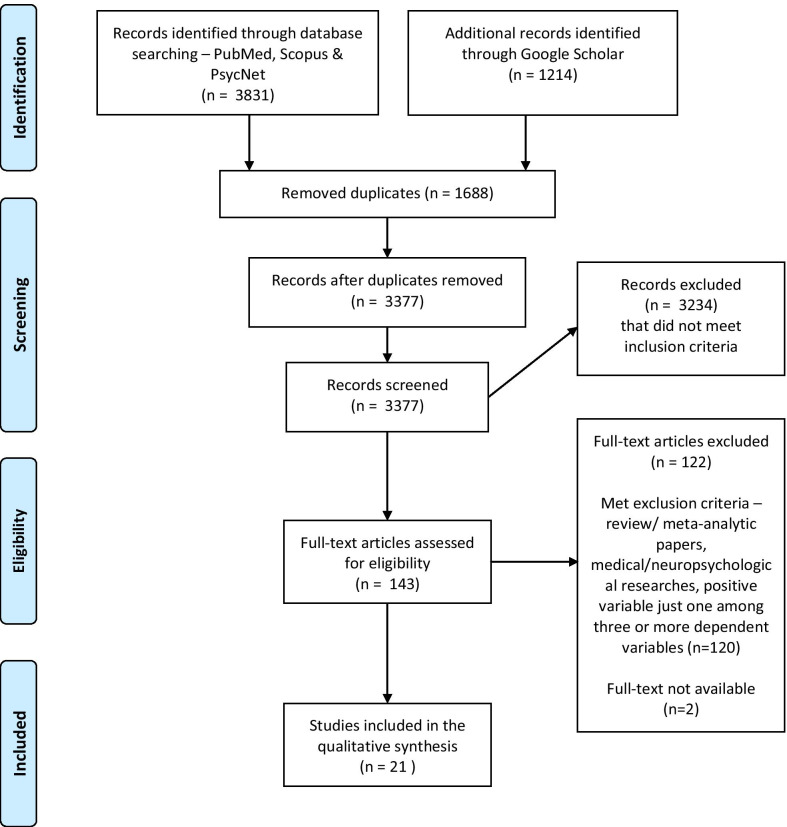
PRISMA flow diagram [65]

A complete database search on PubMed, Scopus, and PsycNet was carried out along with a manual search in Google Scholar (see Fig. 1). From the four electronic databases, 5045 articles were found, whose titles and abstracts were transferred to the reference management software Zotero on 29 May 2020. After elimination of duplicates, 3377 articles remained. The first author had screened the articles and removed 3234 articles that did not meet the inclusion criteria, leaving 143 articles for full-text review. In the list of 143 articles two articles were rejected due to unavailability of full-text. Rest of the 141 articles were scrutinized and 120 articles were removed that met the exclusion criteria—being review/meta-analytic papers, medical/ neuropsychological researches, or studies where positive psychology variables were just one among three or more dependent variables. At the first stage of elimination, the third author had verified 30% of the randomly chosen articles, and at the second stage of elimination the second and third authors had randomly chosen 30% of the full-text articles and cross-verified, after which 100% of consensus was confirmed regarding the exclusion and inclusion of the articles. Finally, 21 articles that reported an MBI with an anticipated impact on positive variables were chosen for the review. Risk of bias tool of Cochrane (2019 version) [36] was used to identify risk of bias of the finally chosen articles. In order to reduce any bias during the process of quality assurance, all of the authors had independently applied the tool among all the chosen studies. Except minor differences of opinion, which were resolved through references to literature and open discussions, no major conflicts had occurred. Studies were found to have low risk or some concerns, and none of the chosen studies had shown high risk.

## Results

Among the 21 articles analyzed, 22 studies were identified, out of which two studies consisted of clinical populations and 20 different normal populations. Collectively, these 22 studies had assessed the impact of the intervention on 134 dependent variables, and 105 of these were positive aspects (eudaimonia and related aspects = 65, hedonia and related variables = 8, general well-being = 7, other positive psychology variables = 25). The finally chosen studies showed a high heterogeneity in terms of research designs, types of intervention, and outcome measures, due to which a narrative/ descriptive synthesis of the data was employed. Table 1 shows the list of studies as indicated by the author name(s) and year of publication, subsequent research designs, sample size, intervention, dependent variables, duration of the intervention, the population to whom the intervention was administered, and the major findings related to the intervention. As per the aim of this review, scope of data analysis is limited to identification of interventions as MPIs or MBIs with expected impact on positive variables. The paper describes the results of the studies as it is reported by the authors. There is scope for further studies to verify the efficacy of the interventions and whether they would produce the positive outcomes they intended to generate or capable of generating.

**Table 1:** The list of studies, corresponding research design, sample size, intervention, dependent variables, duration, population, and major findings

Sl. No	Study	Research design	Sample size	Intervention	Dependent variables	Duration	Population	Major findings
1	Carson et al. [48]	Randomized wait-list controlled design	44 (E = 22, C = 22)	Mindfulness-Based Relationship Enhancement	(i) Relationsi)hip Satisfaction, (ii)Autonomy, (iii)Relatedness, (iv)Closeness, (v)Acceptance of partner, (vi)Relationship distress, (vii)Optimism, (viii)Spirituality, (ix)Individual relaxation, (x) Psychological distress	Eight weekly sessions and a full-day retreat	Happy and non-distressed couples	MBI has beneficially affected all variables assessed, and maintained the effect at a three-month follow-up
2	de Vibe et al. [38]	Longitudinal for six years	288(E = 144, C = 144)	Abridged Mindfulness-Based Stress Programme (MBSR)	(i) Dispositional mindfulness, (ii)Coping, (iii) Well-Being	Seven weeks (15 h) and booster sessions twice yearly	Medical and psychology students	At 6-year follow-up, participants reported better well-being, mindfulness, and problem-focused coping and decreased avoidance focused-coping, irrespective of low intervention adherence
3	Amutio et al. [37]	Longitudinal for one year with randomized controlled design (quasi-experiment)	42 (E = 21, C = 21)	MBSR based on the psycho-educational model of Krasner et al. (2009)	(i) Mindfulness, (ii)Relaxation states, (iii)Heart rate	Eight weeks	Physicians	MBSR has improved mindfulness and relaxation states (including positive emotional states, such as at ease/peace, renewal, energy, optimism, happiness, acceptance, and transcendence) and decreased heart rate. At one-year follow-up effect size improved again
4	Bhayee et al. [46]	Randomized active controlled trial	26 (E = 13, C = 13)	Neurofeedback assisted, technology-supported mindfulness training (NtsMT)	(i) Attention, (ii) Well-being	Six weeks, 10 min of daily practice	Healthy, community-dwelling adults	NtsMT moderately improved attention and well-being
5	Flook et al. [32]	Randomized controlled design	68 (E = 30, C = 38)	Mindfulness-based Kindness curriculum	(i) Social competence, (ii)Sharing, (iii) Delay of gratification, (iv) Cognitive flexibility, (v) Inhibitory control, (vi) Academic performance	12 weeks; 20–30 min sessions twice a week	Pre-school children	Improved social competence, academic performance, delay of gratification, and cognitive flexibility
6	Rasanen et al. [39]	Randomized waitlist controlled design	68 (E = 33, C = 35)	Guided seven-week online Acceptance and Commitment Therapy (iACT)	(i) Well-being, (ii) Life satisfaction, (iii) Self-esteem, (iv) Mindfulness, (v) Stress, (vi) Depression, (vii) Anxiety, (viii)Psychological flexibility, (ix) Sense of coherence	Seven weeks: Two face-to-face meetings and five-weeks iACT	Distressed university students	Well-being, life satisfaction, and mindfulness increased; stress and depression decreased. Benefits were maintained at follow-up after 12 months
7	Fredrickson et al. [53]	Field experiment	139 (E = 67, C = 72)	Loving-Kindness Meditation (LKM)	(i) Mindfulness, (ii) Trait hope, (iii) Savoring beliefs, (iv) Optimism, (v) Ego resilience, (vi) Psychological well-being, (vii) Dyadic adjustment, (viii) Positive relations,, (ix) Illness symptoms, (x) Sleep quality, (xi)Satisfaction with life, (xii) Depression, (xiii)Differential emotions, (xiv) Time varying emotion experiences	Seven weeks: six 60 min group sessions, asked to practice at home, at least 5 days per week, with the guided recordings	Working adults	LKM enhanced experience of positive emotions which benefittedpersonal resources including mindfulness, purpose in life, social support, and illness symptoms. Changes in personal resources predicted improved life satisfaction and reduced depression
8	Yela et al. [52]	Quasi-experimental pre-post design	61 (high adherence = 30, low adherence = 31)	Mindful Self-Compassion	(i) Self Compassion, (ii) Mindfulness, (iii) Well-being, (iv) Anxiety, (iv) Depression	Eight weeks: 2.5 h session once a week	Masters students in clinical and health psychology	High adherence group reported better self-compassion, mindfulness, and well-being
9	Pogrebtsova et al. [56]	daily diary randomized controlled trial	106 (E = 36, C = 36, standard of care C = 34	Combined mindful re-appraisal intervention	(i) Positive and negative experiences, (ii) Positive re-appraisal, (iii) Decentering, (iv) Curiosity, (v) Optimism	Five-day	Undergraduate university students	Experimental group reported reduced negative affect and marginally higher positive affect
10	Smith et al. [59]	Longitudinal quasi experimental design for 2.5-years (two year course and six months follow up)	31 (E = 17, C = 14)	Dharma in Daily Life (DIDL) 30 min per day, six days per week	(I) Quality of life, (ii) Subjective well-being, (iii) Wellbeing, (iv) Valuing, (v) Psychological flexibility, (vi) Mindfulness, (vii) Cognitive fusion	30 min per day, six days a week, for the two year course period and a six month follow-up period	Adults from meditation groups	DIDL improved subjective well-being and mindfulness. Despite the intervention condition, frequency of meditation predicted psychological flexibility, mindfulness, well-being, and valuing
11	Sorensen et al. [54]	Active controlled trial (3 × 3 mixed design)	78 (Convergence = 28, LKM = 26, Music = 24)	Convergence' or LKM or Music for three 2-h group sessions	(1) Mindfulness (2) Self Compassion (3)Fears of Compassion (4) Stress and Anxiety (5) Mental well-being	Three weeks, once weekly	Adults from the general population	All three conditions produced equal benefits on all outcome measures with small effect sizes. No greater impact of Convergence found. Amount of home practice positively correlated with mindfulness and self-compassion at four-week follow-up
12	Devcich et al. [40]	Active controlled pilot study	91 (Mindfulness = 45, Emotional literacy = 46)	Pause, breathe, smile or emotional literacy program	(1) Well-being -hedonia and eudaimonia (2)Mindfulness	One hour weekly sessions for eight weeks	School children	Both conditions improved well-being and only experimental group reported higher mindfulness, post-intervention
13	Ivtzan et al. [58]	Randomized wait-list controlled trial	168 (E = 53, C = 115)	Online Positive Mindfulness Program (PMP)	(1) Eudaimonic and hedonic well-being, (2)Stress, (3)Depression, (4)Mindfulness, (5)Gratitude, (6) Self-compassion, (7) PWB Autonomy, (8) Self efficacy, (9) Meaning in life, (10) Compassion for others, (11)Appreciation for the present moment	Eight-week—12 min audio for daily meditation and 8–10 min video once a week	Citizens from 20 counties, recruited through online forums and social networks	PMP beneficially affected all dependent variables and sustained the effect for 10 out of 11 variables at a one-month follow-up
14	Huppert & Johnson [41]	non-randomized controlled trial	134 (E = 78, C = 56)	Four mindfulness classes	(i) Mindfulness, (ii) Resilience, (iii )Well-being, (iv) Big 5 personality	four 40-min sessions, once a week	14 and 15 year old boys	No between-group difference found. Experimental group reported mindfulness and well-being positively correlated with duration of practice
15	Coatsworth et al. [49]	Randomized controlled comparative effectiveness study design	432 families (E = 154, AC = 160, C = 118)	Mindfulness-Enhanced Strengthening Families Program(	(i) Interpersonal mindfulness in parenting, (ii) Parent-Youth Relationship, (iii) Youth behavior management, (iv) Parent well-being	Once per week for seven weeks	Families of 6th and 7th grade students of four consecutive years	MSFP and a control condition SFP 10–14 showed similar effects on dependent variables. MSFP improved and sustained the effect of SFP on some areas, especially the experience of fathers
16	Vich et al. [57]	Randomized controlled trial	128 (E = 75, C = 42)	Relational Mindfulness Training (RMT)	(i) self compassion, (ii) compassion, (iii) stress, (iv) mindfulness, (v) happiness	Eight week—two hour sessions per week and one six hour session in a weekend	Management students	RMT showed long-term impact on self-compassion, stress, and mindfulness; and short-term impact on compassion and subjective happiness
17	Champion et al. [47]	Pilot randomized controlled trial	62 (E = 29, C = 33)	Headspace app introductory program—Foundation 1 to 3; 30 sessions	(i) Life satisfaction, (ii) Stress, (iii) Resilience, (iv) Social impairment, (v) Depression, (vi) Hypochondriasis, (vii) Anxiety, (viii) Enjoyment & experience	30 sessions with minimum 10 min per session. Option to choose up to 15 to 20 min during level 2 and 3, respectively	Management and economics students	Improved life satisfaction, stress, and resilience. Highest increased on day-10 that dropped moderately by day-30
18	Nyklicek & Kuijpers [28]	Randomized wait-list controlled trial	57	MBSR	(i) Stress, (ii) Vital exhaustion, (iii) Positive affect, (iv) Negative affect, (v) Quality of life, (vi) Mindfulness, (vii) Mindfulness in daily life	Eight weeks—eight weekly sessions of 150 min; and from sixth week an additional six hour session; minimum 40 min of daily practice	Distressed adults	Reduced stress and vital exhaustion, and improved positive affect, quality of life, and mindfulness. Mindfulness, at least partially mediate the impact of MBSR on variables, especially stress, and quality of life
19	Rodriguez-Carvajal etl al. [55]	Non-randomized controlled trial	73 (E = 36, C = 37)	Mindfulness Integrative Model (MIM)	(i) Mindfulness, (ii) Self-compassion, (iii) Positive states of mind	Three weeks—19 sessions	Adults from non-clinical general population	Significant difference in experimental group with large effect size
20	Kappen et al. [50]	Randomized controlled trial	113 (E = 56, C-57)	Online mindfulness program	(i)Relationship satisfaction, (ii) Partner acceptance, (iii) Trait mindfulness	12 day	Adults in a romantic relationship for at least one year recruited through social networking sites	Relationship satisfaction and partner acceptance increased for both groups. Mindfulness significantly improved for low baseline-scorers of experimental condition alone
21	Neff & Germer [51]	Pilot	21	MSC	(i)Self compassion, (ii) Mindfulness, (iii) Connectedness, (iv) Happiness, (v) Life satisfaction, (vi) Depression, (vii) Anxiety, (viii) Stress	Eight weekly sessions and one 2-h session per week	General population recruited through online media	Improved self-compassion, mindfulness, and well-being outcomes
22	Neff & Germer [51]	Randomized controlled trial	52 (E = 25, C = 27)	MSC	(i) Self compassion, (ii) Mindfulness, (iii)Connectedness, (iv)Happiness, (v) Life satisfaction, (vi) Depression, (vii) Anxiety, (viii) Stress, (ix) Compassion for others, (x) Avoidance	Eight weekly sessions and one 2-h session per week	General population recruited through online media	Experimental group reported higher self-compassion, mindfulness, and well-being outcomes, that were maintained at 6-th and one-year follow-ups

E = Experimental group

C = Control group

AC = Active control group

### Interventions and procedures

A brief account of the interventions, research design, and procedures are described in this section. Studies are categorized based on the nature of the interventions, aiming to convey better meaning of the elaborate narration. This section aims to identify the MBIs that have the potential to be an MPI. And the next section, ‘Outcomes of MBIs’, deals with categorizing studies based on the intention of the intervention to enhance eudaimonia or hedonia and other positive variables. Here, depending on the nature of the intervention, they are categorized into eight: (1) Psychotherapies, (2) MBIs for children, (3) Mindfulness apps (4) Positive Relationships, (5) Mindful Self Compassion, (6) Loving Kindness Meditation, (7) MBIs that may act as MPIs and (8) MPIs.

1. Psychotherapies

MBIs are commonly used as therapeutic strategies, even when the positive outcomes are being explored. In such a study, Nyklícek & Kuijpers [28] had applied the MBSR intervention among distressed adults using a randomized waitlist controlled trial. The study intended to find out if the effect of MBSR on stress, vital exhaustion, positive affect, negative affect, quality of life, mindfulness, and daily mindfulness were mediated by mindfulness. Another research carried out by Amutio et al. [37] attempted to estimate the effect of MBSR on mindfulness and relaxation states of 42 physicians. The primary aim of the study was to test the efficacy of MBSR in inducing relaxation among professionals from a highly distressing career background. Also, heart rate was included as a dependent variable in order to confirm that MBSR could act as a relaxation method at a physical level as well. Although relaxation is a byproduct of mindfulness, other possible positive outcomes that could have opened ways to enhance human well-being and flourishing, were not the object of focus in this study. In another study, de Vibe et al. [38] had reported a six-year-long longitudinal study, where the impact of a seven-week abridged MBSR is described. The study illustrates the well-being, coping, and mindfulness of 288 participants. Another popular psychotherapy that makes use of mindfulness is Acceptance and Commitment Therapy (ACT). A guided seven-week internet-delivered Acceptance and Commitment Therapy (iACT) was administered among 68 university students with high distress. The participants’ well-being (psychological, emotional, and social domains), life satisfaction, self-esteem, mindfulness, stress, anxiety, depression, psychological flexibility, and sense of coherence were assessed by eight psychological assessment tools [39]. Since the study had combined clinical and positive outcome measures and because the interaction among these variables was uncertain, it is safe to refrain from concluding that iACT would be useful as a positive psychology intervention. Mostly, the MBIs with psychotherapeutic properties are predominantly governed by deficit-reduction qualities and the positive outcomes are only consequential.

2. MBIs for children

Three of the reviewed studies had been conducted among children [32, 40, 41]. Eudaimonic well-being among children is an area in the scientific literature with extremely less empirical information [42]. And the operational definitions of eudaimonia assessed among children are found to be limited in scope. In a study that attempted to see the impact of an MBI among child population, Huppert & Johnson [41] had administered four 40 minutes of mindfulness classes, one session per week, to 155 boys belonging to the age group of fourteen and fifteen years. Pre and post-assessments were conducted on their mindfulness, resilience, well-being, and big-five personality variables. In another research, Flook et al. [32] had observed 68 preschool children who were administered with a 12-week Mindfulness-Based Kindness Curriculum. A randomized waitlist controlled design was employed to obtain the amount of their social competence (a combination of pro-social behavior and emotion regulation), sharing, delay of gratification, cognitive flexibility, inhibitory control, and academic performance. Devcich et al. [40] had carried out another study where a novel intervention namely “Pause, Breathe, Smile” was tested for its efficacy with an active-controlled pilot design, against an emotional literacy program. Duration for both of the programs was one-hour weekly sessions for eight weeks. The study assessed pre and post scores of 91 school children, on well-being—including hedonia and eudaimonia—and mindfulness. Considering that the target population is children, it is not to be expected to find a concept as complex as eudaimonia to be manipulated or measured effectively, particularly when the interventions do not follow a standardized procedure. Although mindfulness was taught, the studies did not primarily focus on the well-being or other positive functioning of the participants, possibly because of the difficulty in gathering information on positive experiences from children. Hence, the three different interventions adopted here cannot be considered as effective tools for enhancing well-being and flourishing, but they shall be useful tools for specific targeted behavioral modifications and academic performance.

3. Mindfulness apps

The use of online platforms for counselling and psychotherapy is becoming popular nowadays, especially since the outbreak of covid 19 pandemic in 2020 [43, 44]. Not just the reduction of undesirable states of mind, but the enhancement of positive functioning is also getting wide acceptance at a global level [45]. There are a few commercial mindfulness-based applications accessible though smart phones that were also empirically validated through scientific researches. In this review of MBIs, two of the studies chosen had implemented two apps—“Calm” and “Headspace” to explore its impact on health and well-being. Bhayee et al. [46] had tested the therapeutic efficacy of a commercial neurofeedback assisted, technology-supported mindfulness training (NtsMT). The experimental group was exposed to the “Calm” app in a pre-planned manner with recorded instructions. They have used a randomized active-control trial among 26 participants. Electroencephalogram (EEG) was used as the neurofeedback mechanism, and the psychological variables assessed were attention and well-being. Champion et al. [47] had conducted another research using a self-guided mindfulness meditation app, “Headspace”. The introductory program of the Headspace, “Foundation 1 to 3” with 30 sessions (10 at each level), was administered to the participants. The minimum duration of a session was 10 min, and there was an option to increase the duration up to 15 and 20 min for second and third levels respectively. They have assessed the life satisfaction, stress, resilience, social impairment, depression, anxiety, hypochondriasis, and enjoyment and experience of 62 participants. Both of these studies had apparently anticipated reduced clinical symptoms from the interventions, and the range of positive outcomes assessed were too narrow, suggesting that the intended use of these apps, in the concerned studies, was not primarily positive functioning.

4. Positive relationships

*Positive relationships* is a component of eudaimonic well-being. Three studies selected for the review had utilized three different interventions with the principal aim of improving relationships. Carson et al. [48] had tested the effect of a novel intervention, *Mindfulness-Based Relationship Enhancement* (MBRE), on relationship satisfaction, relatedness, autonomy, interpersonal closeness, partner acceptance, relationship distress, spirituality, individual relaxation, and psychological distress. They had adopted a randomized waitlist controlled design and the participants were 44 relatively happy and non-distressed couples. Another MBI that aimed at improving ‘positive relationship’ was applied in a study by Coatsworth et al. [49]. They had tested the efficacy of the *Mindfulness-Enhanced Strengthening Families Program* (MSFP) against a *standard of care condition* and control groups. They have adopted a randomized controlled comparative effectiveness study design with 432 families. The intervention intended to impact interpersonal mindfulness in parenting, parent-youth relationship, youth behavior management, and parent well-being. MSFP was an adapted intervention meant to be a preventive measure to protect adolescents from substance use and behavior problems. With the added element of mindfulness in the adapted version of the intervention, some positive outcomes were also anticipated which were included as dependent variables. Kappen et al. [50] had conducted another study on *positive relationship*, using a brief 12-day online mindfulness program. Intended outcomes of this intervention were elevated relationship satisfaction, partner acceptance, and trait mindfulness. Adults who had been in a romantic relationship for at least one year were recruited through social networking sites. Despite being context-specific and not focusing on relationship enhancement in an exhaustive way, these interventions definitely throw some light on the status of MBIs that are considered to be relationship enhancers. Specifically, MBRE, MSFP, and the 12-day online mindfulness program are apparently effective to improve quality of relationships at specific contexts. Since these interventions are designed for healthier relationship between specific target populations, such as couples, the same interventions will not be sufficient to improve relationship quality in another situation.

5. Mindful self compassion (MSC)

MSC, an intervention developed by Neff and Germer [51], intends to build self compassion in both normal and clinical populations. It is fundamentally a mindfulness-based positive psychology intervention, which gives priority to self-compassion and secondary importance to mindfulness. Other outcomes resultant from compassion and mindfulness shall also be expected from MSC, but its focus is not shared with any further components of well-being or other positive psychology variables. The current review found two papers where three studies that employed MSC were reported. Neff & Germer [51] had performed a pilot study and another randomized waitlist controlled trial to examine the effect of the Mindful Self Compassion (MSC) program. The intervention was for eight weeks, one two-hour session per week. They have studied the impact of the intervention on self-compassion, mindfulness, connectedness, happiness, life satisfaction, depression, anxiety, and stress in the first study and have added two more dependent variables in the second study, which are avoidance, and compassion for others. In another study, Yela et al. [52] had explored the impact of a Mindful Self-Compassion (MSC) program on self-compassion, mindfulness, psychological well-being, anxiety, and depression among 61 psychology trainees. The intervention lasted for eight weeks, with a 2.5-hour session weekly. The MSC interventions applied in these three studies have acted as psychological tools to improve specific elements of eudaimonic and hedonic well-being, along with other factors. The positive impacts of the interventions were looked upon from the point of view of ‘improved well-being through improved mental health’ rather than enhancement of well-being, happiness, flourishing, or meaning in life. It is difficult to conclude whether MSC was effectively established as an MPI through the aforementioned studies, considering the nature of MPI as an intervention with primary focus on positive outcomes. Nevertheless, the study results indeed emphasize the positive potentials of MSC.

6. Loving kindness meditation (LKM)

LKM is a kind of Buddhist meditation that intends to induce “a feeling of warmth and caring for self and others” [53]. Among the 22 studies reviewed, two studies had incorporated interventions that utilized LKM. Fredrickson et al. [53], in their study, recruited 139 working adults into experimental and waitlist control groups and the former was administered with 13 measures that assessed 15 variables—mindfulness, agency thinking, pathway thinking, savoring beliefs, optimism, ego resilience, psychological well-being, dyadic adjustment, positive relations, illness symptoms, sleep quality, satisfaction with life, depression, differential emotions, and emotion experiences. LKM was provided to the former group that extended for seven weeks with one hour weekly sessions. In a different study, Sorensen et al. [54] had investigated the effects of a novel intervention called ‘Convergence’ that combined LKM and classic guitar music. The two active-controlled conditions were given either music alone or meditation alone. All three conditions were prolonged for three weeks, providing one session per week, and the participants were assessed for mindfulness, self-compassion, fears of compassion, stress and anxiety, and mental well-being. Both of these researches focused less on the positive qualities of the intervention. LKM is a meditation practice that involves mindfulness elements but with an additional intentional focus on warm and tender feelings toward oneself and the others. LKM strives to instill an attitude of loving-kindness and do not attempts to enhance any other psychological properties directly. But the study results indicate that it is sufficient to improve specific aspects of hedonic and eudaimonic well-being.

7. MBIs that may act as MPIs

Positive psychology is relatively young and the number of studies is not yet comparable with that of clinical psychology and other deficit-focused fields of psychology. But it is a rapidly developing area that overlaps with the studies on mindfulness. There were three researches in this review where the positive psychological variables were looked into more vigorously. One of these was reported by Rodríguez-Carvajal et al. [55] where a non-randomized controlled study was used among 73 participants to substantiate the effect of a three-week Mindfulness Integrative Model (MIM) on mindfulness, self-compassion, and positive states of mind. In another instance, Pogrebtsova et al. [56] had studied the impact of a five-day combined mindful-reappraisal intervention on students’ positive and negative experiences, positive re-appraisal, decentering, curiosity, and optimism. The sample consisted of 106 participants where 36 were in the experimental group, which was compared against a ‘standard of care’ condition and active control group. The third study was carried out by Vich et al. [57] where a modified intervention, ‘Relational Mindfulness Training’ (RMT) was administered to 75 management students, and their self-compassion, compassion, stress, mindfulness, and happiness were measured. Despite a larger part of well-being aspects being still unexplored, the positive potentials of MBIs are well-documented in these researches. Further studies shall unravel the actual positive qualities of these interventions.

8. Mindfulness-based positive psychology interventions (MPIs)

It sounds as if two studies had explored the exponential positive power of MBIs. Ivtzan et al. [58] had studied the impact of a novel MPI, eight-week online ‘Positive Mindfulness Program’ (PMP) on eudaimonic and hedonic well-being, stress, depression, mindfulness, gratitude, self-compassion, autonomy component of psychological well-being scale, self-efficacy, meaning in life, compassion for others, and appreciation for the present moment, among 168 adults from 20 different countries. They have used a randomized waitlist controlled trial with pre, post, and one-month follow up data. Here, PMP had tested both clinical and positive outcomes, but basically it is an intervention developed to improve well-being through nine specific components –(i) positive emotions, (ii) self-compassion, (iii) well-being (happiness), (iv) autonomy, (v) mindfulness, (vi) self-efficacy (strengths), (vii) meaning, (viii) compassion, and (ix) engagement (savoring)’ [1]. In a different study by Smith et al. [59], 31 meditating adults were assessed for quality of life, subjective well-being, well-being, valuing, psychological flexibility, mindfulness, and cognitive fusion. The experimental group consisted of 17 individuals who had practiced Dharma in Daily Life (DIDL) for 30 minutes per day, extending six days a week, for two years course period and six months follow-up period, and possibly beyond. DIDL indeed had undeniable positive impact, but the intensity and duration raises questions about its feasibility as a common MPI. Nevertheless, both PMP and DIDL show promising utility of MPIs for enhancement of eudaimonia, hedonia, and other specific positive variables.

### Outcomes of MBIs

The reviewed studies vary greatly based on research designs, outcome measures, intensity and structure of interventions, and analytical methods adopted. Hence, due to this high heterogeneity, it was only possible to narrate a peripheral report of outcomes. Depending on the effect sizes of outcome measures, there is an extended scope for further studies which surpass the objectives of the current review. Here, based on the intended positive outcomes of the interventions, studies are categorized into: (1) Enhancement of eudaimonia, (2) Enhancement of hedonia, and (3) Enhancement of other positive variables.

1. Enhancement of eudaimonia

In a randomized controlled efficacy trial, Rasanen et al. [39] found that there is a significant increase in well-being, life satisfaction, and mindfulness among the participants who had exposed to the iACT. They had also reported less stress and depression. These effects were intact in a 12-month follow-up as well. The study results show the plausible impact of an MBI on well-being, life satisfaction, and mindfulness, that shall contribute to a sense of purposeful living, one of the different components of eudaimonia.

Devcich et al. [40] had administered a mindfulness-based intervention, ‘Pause, Breathe, Smile’, to 45 school children as part of a research. Compared to an active control group, the former children had shown higher mindfulness and well-being (hedonia, eudaimonia, and socially desirable responsibility). The MBI ‘Pause, Breathe, Smile’ is likely to contribute to the eudaimonic well-being of children, not in a comprehensive way but to a limited extent.

Carson et al. [48] had observed the significant positive impact of Mindfulness-Based Relationship Enhancement on relationship functioning and well-being of couples, even when the couples were relatively happy and non-distressed at the baseline level. They had received results that supported the beneficial effect of the MBI on all dependent variables that was maintained at a three-month follow-up. In their study, Coatsworth et al. [49] had applied MSFP for strengthening four conditions related to family functioning. They have concluded that MSFP improved interpersonal mindfulness in parenting, parent-youth relationships, youth behavior management, and parent well-being. Kappen et al. [50] reported that, after a 12-day online mindfulness practice, 56 participants with lower baseline mindfulness reported higher relationship satisfaction and partner acceptance compared to the control group. Otherwise, both the groups, regardless of the administration of mindfulness practice or psycho-education, showed no significant difference in the aforementioned variables. In these studies, three different interventions indicate the possible usage of MBIs for ‘positive relationships’, a component of eudaimonic well-being.

Yela et al. [52] had studied the effects of MSC program and found that it has a significant impact on self-compassion, mindfulness, and psychological well-being (PWB) or eudaimonia. Despite focusing on the enhancement of compassion alone, the intervention proved to be a potential MPI for eudaimonic enhancement.

Ivtzan et al. [58] had tested the impact of PMP, on 11 psychological variables. The result indicated that there was a significant difference between the experimental and control groups on the basis of their scores of all the 11 dependent variables, including eudaimonic and hedonic well-being, mindfulness, meaning in life, compassion, and gratitude. PMP focuses on enhancement of both hedonic and eudaimonic well-being and accounts promising outcomes as an MPI for eudaimonic enhancement.

2. Enhancement of hedonia

In a field experimental study, Fredrickson et al. [53] found that the practice of LKM improved participants’ positive emotions (amusement, awe, contentment, joy, gratitude, hope, interest, love, and pride, collectively), and its effect expanded beyond the duration of meditation and cumulated overtime. Pogrebtsova et al. [56] had administered a five-day mindful reappraisal intervention to 36 participants and acquired results that suggest a decrease in negative affect and increase in positive affect toward the end of the intervention, compared to the scores of an active control and a *standard of care* conditions. Smith et al. [59] elucidated an instance when 17 participants were studied against a control group of 14 after getting exposed to DIDL intervention. It was stated that the experimental group, post-intervention, reported higher subjective well-being, well-being, mindfulness, psychological flexibility, and valuing. Nyklicek & Kuijpers [28] had narrated the impact of MBSR on stress, vital exhaustion, positive affect, quality of life, and mindfulness, in a randomized controlled trial. Compared to the control group, individuals exposed to the MBSR reported decreased stress and vital exhaustion, and increased positive affect, quality of life, and mindfulness. In a study using MIM, Rodriguez-Carvajal et al. [55] had found that the intervention enhanced mindfulness, self-compassion, and positive mental states.

The interventions mentioned above are valuable in improving specific aspects of hedonic well-being—either by reducing negative experiences or by improving pleasure and joy.

3. Enhancement of other positive variables

Flook et al. [32] had obtained evidence for a 12-week mindfulness-based Kindness Curriculum being effective in improving social competence, including pro-social behavior and emotion regulation, of pre-school children. It had also improved academic performance, tendency to delay gratification, and cognitive flexibility. In a longitudinal study, de Vibe et al. [38] reported that after a six-year follow-up, the participants who had undergone a 7-week abridged MBSR scored higher in well-being, mindfulness, and problem-focused coping that was a predictor of higher well-being. They had also revealed deteriorated avoidance-focused coping. The results were present even among the participants with low adherence to the regular practice of MBSR. Amutio et al. [37] have also described the effect of MBSR on well-being and related variables. At the end of the intervention period, participants in the experimental group scored significantly higher in mindfulness and relaxation. After a 10-month maintenance phase, their already reported positive outcomes were found to have increased even higher, particularly the scores on mindfulness, and all four dimensions of relaxation state—mindfulness, positive energy, transcendence, and relaxation. Bhayee et al. [46], using the app 'Calm', had studied the impact of an NtsMT on attention and well-being. The result suggested a moderate effect of mindfulness on attention and well-being while previous literature had a different say on its effect size. The reason shall be attributed to the delivery mode of the intervention, its duration, or both. Sorensen et al. [54] had introduced a novel intervention, *Convergence*, that was tested for its efficacy in comparison with an LKM-only group and a music-only group. The results indicated that all these three conditions improved mindfulness, self compassion, and well-being with small effect sizes. A study conducted by Huppert & Johnson [41] revealed high positive association between the time spent for mindfulness practice outside the intervention period and the amount of mindfulness and well-being. Other than that, between control and experimental groups, no significant differences were observed. The effect of RMT on compassion, stress, and mindfulness were assessed by Vich et al. [57]. Their study results outlined that RMT has a significant impact on self-compassion, stress, and mindfulness in the long run. RMT had an impact on compassion, and subjective happiness for a short time, but failed to sustain it over time. In a pilot randomized controlled trial, Champion et al. [47] had received the effect of the use of a mindfulness meditation app ‘Headspace’ on life satisfaction, stress, and resilience. Highest improvement was on 10^th^ session, that dropped moderately by the last and 30th session. Through two subsequent studies, Neff & Germer [51] obtained evidences for the impact of MSC on enhanced mindfulness, self-compassion and well-being.

### MBIs as mindfulness-based positive psychology interventions (MPIs)

In the current systematic review, 21 papers were reviewed that described 22 studies on the impact of MBIs over positive human functioning, with prime importance given to eudaimonic well-being and secondary preference given to hedonic and other positive psychology variables. The latter was given secondary focus as hedonic well-being or the tendency to seek pleasure and avoid pain is mostly associated with clinical symptom reduction and temporary pleasurable experiences, rather than well-being and flourishing. Most of the MBIs reviewed were developed for specific needs not comprehensively focusing on either eudaimonia or hedonia and other positive psychology variables. Interventions administered among children were reported by three studies [32, 40, 41] and all these three have focused on a few specific positive psychology variables which cannot be attributed to an overall enhancement in eudaimonic or hedonic well-being. Three studies [48–50] have focused on a dimension of eudaimonic well-being—positive relationships. One of these is an adapted preventive intervention for adolescent substance use and problem behavior, and could not be considered as an MPI. The target population for six studies was college/university students [38, 39, 47, 52, 56, 57]. One of these has chosen only distressed students and none of the studies focused entirely on well-being. Ten studies had recruited the general adult population through online or regular modes. Some studies advertised for volunteers as participants and some have recruited participants from institutions under different conditions. Most of the studies offered remunerations at various points. None of these studies employed interventions for enhancement of well-being with prime importance, and the positive impact of all of these MBIs was limited, focusing on specific aspects like self-compassion, mindfulness, or resilience. Hence, without ignoring the positive potential of these MBIs, it is required to point out the need for exploring positive outcomes of MBIs more extensively, and modify the existing interventions if required, to incorporate facilities to enhance positive outcomes.

## Limitations

The review was restricted to three databases and manual search, and the possibility of unintentional exclusion of relevant articles indexed in other databases cannot be ignored. It is also possible that some of the excluded articles that primarily focus on clinical variables had reported the positive potentials of those clinical interventions. Though not high, there is a risk of bias in the cumulative result. And high heterogeneity of reviewed studies restricted the current research to opt for a systematic review rather than meta-analysis.

## Conclusions

The current review has identified the major studies where MBIs were applied and its impact on positive human functioning assessed. The nature, pattern, duration, and focal area of interventions varied greatly and mostly centered around a few specific positive variables rather than overall well-being and flourishing. Application of MBIs for hedonic and other positive variables is found to be more frequent than the usage of MBIs for eudaimonic enhancement. This was not concluded from just the review of the final 21 articles, but from the entire process of finalizing those studies. This is consistent with the statement of Deci & Ryan [60] who had noted that the number of studies on hedonia greatly exceeds than that on eudaimonia. Hedonic well-being was closely associated with clinical symptom reduction instead of increment in the experience of perceived pleasure. Most of the MBIs applied with expected positive outcomes were context-specific or limited in the scope of applicability. The review was futile in finding any singularly positive-psychology oriented interventions, but a few of the interventions show powerful utility as an MBI that could enhance *specific* positive variables. Further empirical explorations shall reveal the potency of these MBIs as mindfulness-based positive psychology interventions. Modifications in the structure and functions to be more inclusive of contexts and populations would yield better positive outcomes of the existing MBIs. Also, MBIs that aimed at catering the needs of the recipients based on factors such as culture, ethnicity, and gender would result in highly effective MPIs. From the review, it could also be concluded that it is imperative to develop interventions with sole focus on enhancement of positive potentials, especially eudaimonic enhancement.

Study results point out that physical pleasures derived out of hedonia are not sufficient for the experience of well-being [61, 62, 63]. Keyes & Annas [64] pointed out the gulf between individuals with high hedonic well-being (48.5%) and their flourishing (18%). This explains the severe eudaimonic deficiency that contributes to the lack of flourishing. And in some other personal or social situations where hedonia can contribute little to a person’s well-being—such as chronic illnesses, physical or psychological pain, financial insecurity, childlessness, bereavement, or social/political unrest—eudaimonia is inevitable to maintain general well-being, happiness, contentment, and a sense of meaning and purpose in life. Eudaimonia apparently buffers against possible psychological harm also [64]. Hence it demands explorations in the direction of eudaimonic enhancement across different populations, cultures, and contexts. Unfortunately, few researches have addressed this issue so far and eudaimonic enhancement still remains a neglected area within applied positive psychology. Considering the paucity of MPIs exclusively for eudaimonic enhancement, it is recommended that immediate further actions are essential to develop, validate, and avail the same, among both clinical and non-clinical populations. In conclusion, the current study has reviewed the major studies where the MBIs are used for enhancement of eudaimonia, hedonia, and other positive psychology variables. It contributes to the existing scientific literature by pointing out the positive potentials of MBIs and the endless possibilities of empirical studies on the application of MPIs. Finally, the review emphasizes the need of future studies paying attention to the utilization of eudaimonic enhancement potential of MPIs along with the focus on enhancement of hedonic and other positive outcomes.

## Data Availability

The datasets used and/or analysed during the current study are available from the corresponding author on reasonable request.

## Abbreviations

MBI: Mindfulness-Based Intervention
PPI: Positive Psychology Intervention
MPI: Mindfulness-Based Positive Psychology Intervention
MBSR: Mindfulness Based Stress Reduction
iACT: Internet-delivered Acceptance and Commitment Therapy
NtsMT: Neurofeedback assisted technology supported mindfulness training
EEG: Electroencephalogram
MBRE: Mindfulness-Based Relationship Enhancement
MSFP: Mindfulness-Enhanced Strengthening Families Program
MSC: Mindful Self Compassion
LKM: Loving Kindness Meditation
MIM: Mindfulness Integrative Model
RMT: Relational Mindfulness Training
PMP: Positive Mindfulness Program
DIDL: Dharma in Daily Life

## References

[1] Lomas T, Ivtzan I. Beyond deficit reduction: exploring the positive potentials of mindfulness. In: Shonin E, Gordon WV, Griffiths MD, editors. Mindfulness and buddhist-derived approaches in mental health and addiction. Cham: Springer International Publishing; 2016. pp. 277–95. 10.1007/978-3-319-22255-4_14.

[2] Kabat-Zinn J. Full catastrophe living: using the wisdom of your body and mind to face stress, pain, and illness. New York, NY: Bantam Dell; 2005.

[3] Toniolo-Barrios M, Pitt L (2021). Mindfulness and the challenges of working from home in times of crisis. Bus Horiz.

[4] Stankov U, Filimonau V, Vujičić MD (2020). A mindful shift: an opportunity for mindfulness-driven tourism in a post-pandemic world. Tour Geogr.

[5] Reina CS, Kudesia RS (2020). Wherever you go, there you become: How mindfulness arises in everyday situations. Organ Behav Hum Decis Process.

[6] Conversano C, Di Giuseppe M, Miccoli M, Ciacchini R, Gemignani A, Orrù G (2020). Mindfulness, age and gender as protective factors against psychological distress during COVID-19 pandemic. Front Psychol.

[7] Shankland R, Tessier D, Strub L, Gauchet A, Baeyens C (2021). Improving mental health and well-being through informal mindfulness practices: an intervention study. Appl Psychol Health Well Being.

[8] Feldman O, Goldstien E, Rolnik B, Ganz AB, Lev-Ari S (2021). Inquiry based stress reduction (IBSR) improves overall stuttering experience among adults who stutter: a randomized controlled trial. J Clin Med.

[9] Zadok-Gurman T, Jakobovich R, Dvash E, Zafrani K, Rolnik B, Ganz AB (2021). Effect of inquiry-based stress reduction (IBSR) intervention on well-being, resilience and burnout of teachers during the COVID-19 pandemic. Int J Environ Res Public Health.

[10] Long R, Halvorson M, Lengua LJ. A mindfulness-based promotive coping program improves well-being in college undergraduates. Anxiety, Stress, & Coping. 2021;0:1–14. 10.1080/10615806.2021.1895986 33719757

[11] Layous K, Chancellor J, Lyubomirsky S, Wang L, Doraiswamy PM (2011). Delivering happiness: translating positive psychology intervention research for treating major and minor depressive disorders. J Altern Complem Med.

[12] Sin NL, Lyubomirsky S (2009). Enhancing well-being and alleviating depressive symptoms with positive psychology interventions: a practice-friendly meta-analysis. J Clin Psychol.

[13] Fava GA, Rafanelli C, Cazzaro M, Conti S, Grandi S (1998). Well-being therapy. A novel psychotherapeutic approach for residual symptoms of affective disorders. Psychol Med.

[14] Fava GA, Ruini C, Rafanelli C, Finos L, Salmaso L, Mangelli L (2005). Well-being therapy of generalized anxiety disorder. Psychother Psychosom.

[15] Steck EL, Abrams LM, Phelps L (2004). Positive psychology in the prevention of eating disorders. Psychol Schs.

[16] Huta V. Eudaimonia versus Hedonia: What Is the Difference? And Is It Real? | International Journal of Existential Positive Psychology. In: Special Issue: Proceedings of the 2016 Meaning Conference. International Journal of Existential Positive Psychology; 2018. p. 8. http://journal.existentialpsychology.org/index.php/ExPsy/article/view/230. Accessed 20 Jul 2020.

[17] Ryff CD, Dienberg Love G, Urry HL, Muller D, Rosenkranz MA, Friedman EM (2006). Psychological well-being and ill-being: do they have distinct or mirrored biological correlates?. Psychother Psychosom.

[18] Waterman AS, Schwartz SJ, Zamboanga BL, Ravert RD, Williams MK, Agocha VB (2010). The Questionnaire for Eudaimonic well-being: psychometric properties, demographic comparisons, and evidence of validity. J Posit Psychol.

[19] Huta V (2013). Eudaimonia in oxford handbook of happiness.

[20] Garland EL, Farb NA, Goldin P, Fredrickson BL (2015). Mindfulness Broadens awareness and builds eudaimonic meaning: a process model of mindful positive emotion regulation. Psychol Inq.

[21] Waterman AS (2007). On the importance of distinguishing hedonia and eudaimonia when contemplating the hedonic treadmill. Am Psychol.

[22] Fredrickson BL (2004). The broaden–and–build theory of positive emotions. Phil Trans R Soc Lond B.

[23] Chang J-H, Huang C-L, Lin Y-C (2015). Mindfulness, basic psychological needs fulfillment, and well-being. J Happiness Stud.

[24] Wolsko C, Lindberg K (2013). Experiencing connection with nature: The matrix of psychological well-being, mindfulness, and outdoor recreation. Ecopsychology.

[25] Hong PY, Lishner DA, Han KH (2014). Mindfulness and eating: an experiment examining the effect of mindful raisin eating on the enjoyment of sampled food. Mindfulness.

[26] Malboeuf-Hurtubise C, Taylor G, Lefrançois D, Essopos I, Lacourse E (2018). The impact of a mindfulness-based intervention on happiness: a reflection on the relevance of integrating a positive psychology framework within mindfulness research in youth. Int J Appl Posit Psychol.

[27] Howells A, Ivtzan I, Eiroa-Orosa FJ (2016). Putting the ‘app’ in happiness: a randomised controlled trial of a smartphone-based mindfulness intervention to enhance wellbeing. J Happiness Stud.

[28] Nyklícek I, Kuijpers KF (2008). Effects of mindfulness-based stress reduction intervention on psychological well-being and quality of life: is increased mindfulness indeed the mechanism?. Ann Behav Med.

[29] Munoz RT, Hoppes S, Hellman CM, Brunk KL, Bragg JE, Cummins C (2018). The effects of mindfulness meditation on hope and stress. Res Soc Work Pract.

[30] Heckenberg RA, Hale MW, Kent S, Wright BJ (2019). An online mindfulness-based program is effective in improving affect, over-commitment, optimism and mucosal immunity. Physiol Behav.

[31] Schonert-Reichl KA, Lawlor MS (2010). The effects of a mindfulness-based education program on pre- and early adolescents’ well-being and social and emotional competence. Mindfulness.

[32] Flook L, Goldberg SB, Pinger L, Davidson RJ (2015). Promoting prosocial behavior and self-regulatory skills in preschool children through a mindfulness-based Kindness Curriculum. Dev Psychol.

[33] Scott-Hamilton J, Schutte NS, Brown RF (2016). Effects of a mindfulness intervention on sports-anxiety, pessimism, and flow in competitive cyclists. Appl Psychol Health Well Being.

[34] Quach D, Jastrowski Mano KE, Alexander K (2016). A randomized controlled trial examining the effect of mindfulness meditation on working memory capacity in adolescents. J Adolesc Health.

[35] Franco C, Mañas I, Cangas AJ, Gallego J. The Applications of Mindfulness with Students of Secondary School: Results on the Academic Performance, Self-concept and Anxiety. In: Lytras MD, Ordonez De Pablos P, Ziderman A, Roulstone A, Maurer H, Imber JB, editors. Knowledge Management, Information Systems, E-Learning, and Sustainability Research. Berlin, Heidelberg: Springer; 2010. p. 83–97.

[36] Risk of bias tools - Current version of RoB 2. https://sites.google.com/site/riskofbiastool/welcome/rob-2-0-tool/current-version-of-rob-2. Accessed 16 Dec 2020.

[37] Amutio A, Martínez-Taboada C, Hermosilla D, Delgado LC (2015). Enhancing relaxation states and positive emotions in physicians through a mindfulness training program: a one-year study. Psychol Health Med.

[38] de Vibe M, Solhaug I, Rosenvinge JH, Tyssen R, Hanley A, Garland E (2018). Six-year positive effects of a mindfulness-based intervention on mindfulness, coping and well-being in medical and psychology students; Results from a randomized controlled trial. PLoS ONE.

[39] Räsänen P, Lappalainen P, Muotka J, Tolvanen A, Lappalainen R (2016). An online guided ACT intervention for enhancing the psychological wellbeing of university students: a randomized controlled clinical trial. Behav Res Ther.

[40] Devcich DA, Rix G, Bernay R, Graham E (2017). Effectiveness of a mindfulness-based program on school children’s self-reported well-being: a pilot study comparing effects with an emotional literacy program. J Appl Sch Psychol.

[41] Huppert FA, Johnson DM (2010). A controlled trial of mindfulness training in schools: the importance of practice for an impact on well-being. J Posit Psychol.

[42] Forrest CB, Bevans KB, Filus A, Devine J, Becker BD, Carle AC (2019). Assessing children’s eudaimonic well-being: the PROMIS pediatric meaning and purpose item banks. J Pediatr Psychol.

[43] MacMullin K, Jerry P, Cook K (2020). Psychotherapist experiences with telepsychotherapy: pre COVID-19 lessons for a post COVID-19 world. J Psychother Integr.

[44] Poletti B, Tagini S, Brugnera A, Parolin L, Pievani L, Ferrucci R, et al. Telepsychotherapy: a leaflet for psychotherapists in the age of COVID-19. A review of the evidence. Counselling Psychology Quarterly. 2020;0:1–16.

[45] Huberty J, Green J, Glissmann C, Larkey L, Puzia M, Lee C. Efficacy of the mindfulness meditation mobile app “calm” to reduce stress among College students: randomized controlled trial. JMIR Mhealth Uhealth. 2019;7:e14273. 10.2196/14273 PMC6614998 31237569

[46] Bhayee S, Tomaszewski P, Lee DH, Moffat G, Pino L, Moreno S (2016). Attentional and affective consequences of technology supported mindfulness training: a randomised, active control, efficacy trial. BMC Psychol.

[47] Champion L, Economides M, Chandler C (2018). The efficacy of a brief app-based mindfulness intervention on psychosocial outcomes in healthy adults: A pilot randomised controlled trial. PLoS ONE.

[48] Carson JW, Carson KM, Gil KM, Baucom DH (2004). Mindfulness-based relationship enhancement. Behav Ther.

[49] Coatsworth JD, Duncan LG, Nix RL, Greenberg MT, Gayles JG, Bamberger KT (2015). Integrating mindfulness with parent training: effects of the mindfulness-enhanced strengthening families program. Dev Psychol.

[50] Kappen G, Karremans JC, Burk WJ (2019). Effects of a short online mindfulness intervention on relationship satisfaction and partner acceptance: the moderating role of trait mindfulness. Mindfulness.

[51] Neff KD, Germer CK (2013). A pilot study and randomized controlled trial of the mindful self-compassion program. J Clin Psychol.

[52] Yela JR, Gómez-Martínez MÁ, Crego A, Jiménez L (2020). Effects of the Mindful Self-Compassion programme on clinical and health psychology trainees’ well-being: a pilot study. Clin Psychol.

[53] Fredrickson BL, Cohn MA, Coffey KA, Pek J, Finkel SM (2008). Open hearts build lives: positive emotions, induced through loving-kindness meditation, build consequential personal resources. J Pers Soc Psychol.

[54] Sorensen S, Steindl SR, Dingle GA, Garcia A (2019). Comparing the effects of loving-kindness meditation (LKM), music and LKM plus music on psychological well-being. J Psychol.

[55] Rodríguez-Carvajal R, García-Rubio C, Paniagua D, García-Diex G, De Rivas S (2016). Mindfulness integrative model (MIM): Cultivating positive states of mind towards oneself and the others through mindfulness and self-compassion. Anales de Psicologia.

[56] Pogrebtsova E, Craig J, Chris A, O’Shea D, González-Morales MG (2018). Exploring daily affective changes in university students with a mindful positive reappraisal intervention: a daily diary randomized controlled trial. Stress Health.

[57] Vich M, Lukeš M, Burian J (2020). Out of sight, out of mind? Exploring the long-term effects of Relational Mindfulness Training (RMT). J Contextual Behav Sci.

[58] Ivtzan I, Young T, Martman J, Jeffrey A, Lomas T, Hart R (2016). Integrating mindfulness into positive psychology: a randomised controlled trial of an online positive mindfulness program. Mindfulness.

[59] Smith BM, Ong CW, Barrett TS, Bluett EJ, Slocum TA, Twohig MP (2019). Longitudinal effects of a 2-year meditation and buddhism program on well-being, quality of life, and valued living. Mindfulness.

[60] Deci EL, Ryan RM (2008). Hedonia, eudaimonia, and well-being: an introduction. J Happiness Stud.

[61] Aknin LB, Norton MI, Dunn EW (2009). From wealth to well-being? Money matters, but less than people think: The Journal of Positive Psychology: Vol 4, No 6. J Posit Psychol.

[62] Howell RT, Hill G (2009). The mediators of experiential purchases: Determining the impact of psychological needs satisfaction and social comparison. J Posit Psychol.

[63] Kashdan TB, Breen WE (2007). Materialism and diminished well–being: experiential avoidance as a mediating mechanism. J Soc Clin Psychol.

[64] Keyes CLM, Annas J (2009). Feeling good and functioning well: distinctive concepts in ancient philosophy and contemporary science. J Posit Psychol.

[65] Moher D, Liberati A, Tetzlaff J, Altman DG, Group TP (2009). Preferred reporting items for systematic reviews and meta-analyses: the PRISMA statement. PLOS Med.

